# The burden of premature mortality in Poland analysed with the use of standard expected years of life lost

**DOI:** 10.1186/s12889-015-1487-x

**Published:** 2015-02-07

**Authors:** Irena Maniecka-Bryła, Marek Bryła, Paweł Bryła, Małgorzata Pikala

**Affiliations:** Department of Epidemiology and Biostatistics, Chair of Social and Preventive Medicine, Medical University of Lodz, Żeligowskiego 7/9, Lodz, Poland; Department of Social Medicine, Chair of Social and Preventive Medicine, Medical University of Lodz, Żeligowskiego 7/9, Lodz, Poland; Department of International Marketing and Retailing, University of Lodz, Narutowicza 59a, Lodz, Poland

**Keywords:** Standard expected years of life lost, Premature mortality, Burden of disease, Poland

## Abstract

**Background:**

Despite positive changes in the health of the population of Poland, compared to the EU average, the average life expectancy in 2011 was 5 years shorter for males and 2.2 years shorter for females. The immediate cause is the great number of premature deaths, which results in years of life lost in the population. The aim of the study was to identify the major causes of years of life lost in Poland.

**Methods:**

The analysis was based on a database of the Central Statistical Office of Poland, containing information gathered from 375,501 death certificates of inhabitants of Poland who died in 2011. The SEYLL_p_ (Standard Expected Years of Life Lost per living person) and the SEYLL_d_ (SEYLL per death) measures were calculated to determine years of life lost.

**Results:**

In 2011, the total number of years of life lost by in Polish residents due to premature mortality was 2,249,213 (1,415,672 for males and 833,541 for females). The greatest number of years of life lost in males were due to ischemic heart disease (7.8 per 1,000), lung cancer (6.0), suicides (6.6), cerebrovascular disease (4.6) and road traffic accidents (5.4). In females, the factors contributing to the greatest number of deaths were cerebrovascular disease (3.8 per 1,000), ischemic heart disease (3.7), heart failure (2.7), lung cancer (2.5) and breast cancer (2.3). Regarding the individual scores per person in both males and females, the greatest death factors were road traffic accidents (20.2 years in males and 17.1 in females), suicides (17.4 years in males and 15.4 in females) and liver cirrhosis (12.1 years in males and 11.3 in females).

**Conclusions:**

It would be most beneficial to further reduce the number of deaths due to cardiovascular diseases, because they contribute to the greatest number of years of life lost. Moreover, from the economic point of view, the most effective preventative activities are those which target causes which result in a large number of years of life lost at productive age for each death due to a particular reason, i.e. road traffic accidents, suicides and liver cirrhosis.

## Background

The economic transformation which began in Poland in 1989 substantially influenced the lifestyle of Polish society and its health behaviours [1-4]. Improvements in health caused by the development of new medical technologies and modern diagnostic methods has had an influence on a range of health aspects, including decreasing the mortality rate, which in turn, has led to an increase in average life expectancy. The lifespan of the population of Poland has been systematically increasing since 1991. In 2011, the average life expectancy was 72.4 years for males and 80.9 years for females. In 1990–2011, the values for average lifespan increased by 6.2 years for males and 5.7 years for females [5]. Despite these positive changes, the health condition of the population of Poland in terms of lifespan is much worse than those observed in most European countries. Poland lies in the third ten of a group of 47 countries examined by UNECE, with the males in 30^th^ position and females 27^th^ [6]. According to WHO estimates, the lifespan of a male living in Poland is on average 5 years shorter than that of the mean male lifespan within the European Union as a whole, and 7.5 years shorter than that of males in Sweden, whose lifespan is the longest in the EU. Currently, the average life expectancy for men in Poland is equal to the mean value observed for the European Union 17 years ago. In women these differences are smaller. The lifespan of Polish women is on average 2.2 years shorter than that of women living in the European Union and 4.8 years shorter than that of women from Spain and France, whose lifespan is the longest. The average lifespan in Poland is the same as the mean lifespan observed throughout the European Union 11 years ago [7].

Assuming that all deaths before the age 65 are premature, premature deaths comprised 19% of the total number of deaths in the European Union in 2011, with the corresponding value being 30% in Poland [8]. An immediate result of premature mortality is the number of years lost. It is becoming more common to calculate mortality in units of lost time, as these measurements are more reliable atrevealing the economic and social impact of loss connected with premature mortality. From the economic point of view, the most effective preventative activities are those which aim at reducing the greatest number of years of life lost.

The aim of the study is to identify the factors which contributed to the greatest loss of years per 1,000 inhabitants of Poland, and per individual, in 2011.

## Methods

The research project was granted approval by the Bioethics Committee of the Medical University of Lodz on 22 May 2012 No. RNN/422/12/KB.

A review was performed of information gathered from the death certificates of inhabitants of Poland who died in 2011 (375,501 certificates, including 198.178 men and 177.323 women). All information was obtained from a database maintained by the Department of Information, Central Statistical Office of Poland. Data on population number are based on the National Census of Population and Homes carried out in Poland in 2011.

Years of life lost were counted and analyzed according to Murray and Lopez [9]. The SEYLL (Standard Expected Years of Life Lost) measure was used to calculate the number of years of life lost by the studied population in comparison with the years lost by a referential (standard) population. A mortality standard norm was applied based on the Coale-Demeny west model life table, which has a life expectancy at birth of 80 years for males and 82.5 years for females [10]. For a population of size N, with d_xc_ representing the number of deaths at the age of *x* due to a particular cause *c*, *e*_x_ would be the number of expected years of life that remain to be lived by a population which is at the age of *x*. Assuming that *l* is the last year of age to which the population lives, the number of years of life lost due to cause *c* is calculated with the use of the following formula:

$$ SEYLL={\displaystyle \sum_{x=0}^l{d}_{xc}}{e}_x $$

The average number of years of life lost by one person who died due to cause *c* can be obtained by dividing the absolute number of years lost due to cause *c*, calculated according to the following formula, by the number of deaths due to cause *c*.

$$ SEYL{L}_d=\frac{{\displaystyle \sum_{x=0}^l{d}_{xc}{e}_x}}{{\displaystyle \sum_{x=0}^l{d}_{xc}}} $$

The SEYLL_p_ indices determined by the size of the studied population were also estimated [11,12].

$$ SEYL{L}_p=\frac{{\displaystyle \sum_{x=0}^l{d}_{xc}{e}_x}}{N} $$

The number of years lost due to premature mortality were calculated using 3% time-discounting and age-weighting. The causes of death are classified according to the WHO ICD-10 (Tenth Revision of the International Statistical Classification of Diseases and Health Related Problems). The original Global Burden of Disease Study classified disease and injury causes using a tree structure. The first level of disaggregation comprised three broad cause groups: Group I comprising communicable diseases and maternal, perinatal and nutritional disorders, Group II being chronic non-communicable diseases, and Group III being all injuries. Each group is divided into major subcategories. Beyond this level, there are two further disaggregation levels [13].

## Results

In 2011, the total number of years of life lost due to premature mortality by the inhabitants of Poland was 2,249,213 (1,415,672 for males and 833,541 for females: Table 1), which represents 58.4 years per 1,000 inhabitants (75.9 per 1,000 males and 41.9 years per 1,000 females). The number of lost years of life per single death (SEYLL_d_) was 6.0 (7.1 per males and 4.7 per females). Deaths due to Group II causes contributed to the greatest number of years of life lost. Chronic non-communicable diseases caused 73.6% of the total lost years of life in males (55.8 per 1,000) and 87.4% in females (36.6 per 1,000). However, the number of deaths due to Group III causes, i.e. injuries, varied considerably between the genders: 21.2% of the total number of years of life lost in males and 7.0% in females (the SEYLL measure was 16.1 per 1,000 males and 2.9 per 1,000 females). Deaths due to Group I causes contributed to slightly more than 5% of the total number of years of life lost (the SEYLL_p_ measure was 3.9 per 1,000 males and 2.4 per 1,000 females).

**Table 1:** **Standard expected years of life lost (SEYLL) by sex and three broad cause group, Poland, 2011**

**Cause group**	**Males**			**Females**			**Total**		
	**SEYLL**	**SEYLL**_p_ **per 1,000**	**%**	**SEYLL**	**SEYLL**_p_ **per 1,000**	**%**	**SEYLL**	**SEYLL**_p_ **per 1,000**	**%**
Group I	72961.2	3.9	5.2	46836.8	2.4	5.6	119798.1	3.1	5.3
Group II	1041554.1	55.8	73.6	728125.7	36.6	87.4	1769679.8	45.9	78.7
Group III	301156.6	16.1	21.3	58579.0	2.9	7.0	359735.6	9.3	16.0
**Total**	**1415671.9**	**75.9**	**100.0**	**833541.5**	**41.9**	**100.0**	**2249213.4**	**58.4**	**100.0**

Group I: Communicable, maternal, perinatal and nutritional conditions.

Group II: Non-communicable diseases.

Group III: Injuries.

**Figure 1:**
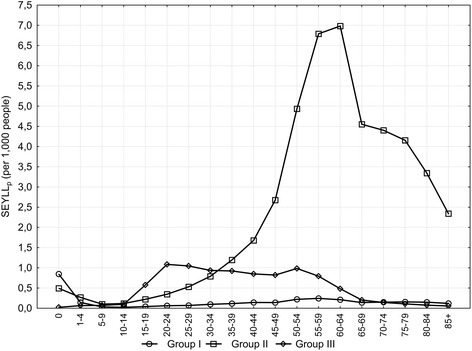
**SEYLL**_p_ **rates by three broad cause groups and age-group, Poland, 2011.**

**Figure 2:**
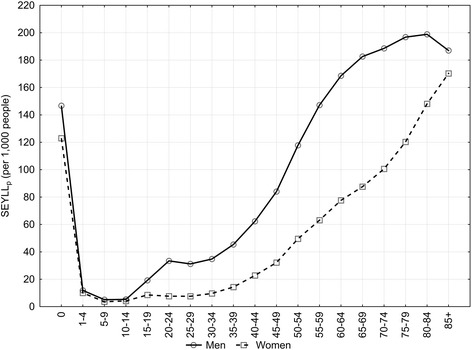
**SEYLL**_p_ **rates by sex and age-group at death, Poland, 2011.**

The primary causes of number of years of life lost vary with regard to age at death. Deaths due to Group I contribute to the greatest number of lost years in the youngest age group, while causes from Group II are most prevalent in the 15 to 34 age group, and causes from Group III for those aged 35 and older (Figure 1). In particular since the age of 15, in all subsequent age groups, SEYLL_p_ are higher for males than females (Figure 2).

With regard to the main causes of lost years of life for males, the greatest number of years were lost to cardiovascular diseases (24.2 per 1,000), malignant neoplasms (19.2 per 1,000), unintentional injuries (10.9 per 1,000), intentional injuries (5.3 per 1,000) and digestive diseases (5.1 per 1,000) (Table 2). Similarly, the greatest number of lost years of life among females were caused by cardiovascular diseases (14.9 per 1,000) and malignant neoplasms (14.7 per 1,000). More distant positions are occupied by digestive diseases (2.3 per 1,000), unintentional injuries (2.2 per 1,000) and perinatal and infant diseases (1.4 per 1,000).

**Table 2:** **Standard expected years of life lost (SEYLL) by sex and main group, Poland, 2011**

**Cause categories**	**Males**				**Females**			
	**SEYLL**	**SEYLL**_p_ **per 1,000**	**%**	**Rank**	**SEYLL**	**SEYLL**_p_ **per 1,000**	**%**	**Rank**
Cardiovascular diseases	451330.7	24.2	31.9	1	296627.1	14.9	35.6	1
Malignant tumors	358135.6	19.2	25.3	2	291951.4	14.7	35.0	2
Unintentional injuries	202599.0	10.9	14.3	3	44526.4	2.2	5.3	4
Intentional injuries	98557.6	5.3	7.0	4	14052.7	0.7	1.7	9
Digestive diseases	94725.0	5.1	6.7	5	44872.8	2.3	5.4	3
Mental and neurological conditions	42068.0	2.3	3.0	6	21446.0	1.1	2.6	6
Perinatal and infant causes	34096.2	1.8	2.4	7	26863.3	1.4	3.2	5
Respiratory diseases	33612.4	1.8	2.4	8	16966.5	0.9	2.0	8
Respiratory infections	32912.0	1.8	2.3	9	21006.7	1.1	2.5	7
Infectious and parasitic diseases	19562.2	1.0	1.4	10	11032.4	0.6	1.3	10

A detailed analysis carried out with consideration of single disease entities indicates that males lose the greatest number of years of life due to ischemic heart disease (7.8 per 1,000) and lung cancer (6.0 per 1,000). In 2011, in Poland, suicides occupied the third position (5.0 per 1,000) for males, followed by cerebrovascular diseases (4.6 per 1,000), road traffic accidents (4.1 per 1,000), heart failure (4.0 per 1,000), liver cirrhosis (2.9 per 1,000) and diseases of the arteries, arterioles and capillaries (mainly including atherosclerosis) (2.0 per 1,000). Among women, the greatest number of years of life lost were caused by cerebrovascular disease (3.8 per 1,000), ischemic heart disease (3.7 per 1,000), heart failure (2.7 per 1,000), lung cancer (2.5 per 1,000), breast cancer (2.3 per 1,000), diseases of the arteries, arterioles and capillaries (2.0 per 1,000) and ovarian cancer (1.1 per 1,000).

However, the causes of the greatest number of lost years of life per single death (SEYLL_d_) are slightly different. According to this criterion, the most significant causes of the loss of years of life for both males and females are road traffic accidents (20.2 years per one male death and 17.1 years per one female death), suicides (17.4 years per male and 15.4 years per female) and liver cirrhosis (12.1 years per male and 11.3 years per female). While cardiovascular diseases contribute to the greatest number of lost years of life per 1,000 people, they occupy more distant positions when the SEYLL_d_ measure is taken into consideration: ischemic heart disease occupies 11^th^ position in males and 17^th^ position in females, while cerebrovascular diseases occupy 13^th^ position in males and 16^th^ position in females. Table 3 presents more detailed data on the indices of years of life lost due to single disease entities which contribute to the greatest number of lost years.

**Table 3:** **Standard expected years of life lost (SEYLL) by sex and single disease entity, Poland, 2011**

**Specific subcategories**	**SEYLL**	**%**	**SEYLL**_p_ **per 1,000**	**Rank**	**SEYLL**_d_	**Rank**
**Males**						
	145678.3	10.3	7.8	1	5.9	11
Lung cancer	112036.7	7.9	6.0	2	7.0	8
Suicides	93374.3	6.6	5.0	3	17.4	2
Cerebrovascular disease	85820.4	6.1	4.6	4	5.6	13
Road traffic accidents	76901.7	5.4	4.1	5	20.2	1
Heart failure	75266.9	5.3	4.0	6	5.3	14
Cirrhosis of the liver	53802.5	3.8	2.9	7	12.1	3
Diseases of arteries, arterioles and capillaries	38066.4	2.7	2.0	8	3.4	17
Influenza and pneumonia	32598.6	2.3	1.7	9	6.1	10
Stomach cancer	23643.3	1.7	1.3	10	6.8	9
Chronic lower respiratory diseases	23437.2	1.7	1.3	11	4.5	15
Colorectal cancer	20975.6	1.5	1.1	12	5.7	12
Prostate cancer	17073.6	1.2	0.9	13	4.2	16
Pancreas cancer	16829.8	1.2	0.9	14	7.5	6
Brain cancer	14985.9	1.1	0.8	15	10.7	4
Leukaemias	12092.9	0.9	0.6	16	7.9	5
Liver cancer	7119.0	0.5	0.4	17	7.1	7
**Females**						
Cerebrovascular disease	74748.5	9.0	3.8	1	3.7	16
Ischaemic heart disease	73338.1	8.8	3.7	2	3.4	17
Heart failure	53653.6	6.4	2.7	3	3.1	18
Lung cancer	49384.8	5.9	2.5	4	7.9	8
Breast cancer	45252.3	5.4	2.3	5	8.3	7
Diseases of arteries, arterioles and capillaries	39207.8	4.7	2.0	6	2.2	19
Ovariancancer	21990.0	2.6	1.1	7	8.6	6
Influenza and pneumonia	20728.4	2.5	1.0	8	4.4	15
Cirrhosis of the liver	20703.9	2.5	1.0	9	11.3	3
Colorectal cancer	18673.1	2.2	0.9	10	5.8	13
Road traffic accidents	17578.1	2.1	0.9	11	17.1	1
Cervix uteri cancer	16829.6	2.0	0.8	12	10.2	4
Pancreas cancer	13617.4	1.6	0.7	13	6.2	11
Brain cancer	12009.8	1.4	0.6	14	9.0	5
Chronic lower respiratory diseases	11835.1	1.4	0.6	15	4.8	14
Suicides	11677.1	1.4	0.6	16	15.4	2
Stomach cancer	10940.4	1.3	0.6	17	6.2	10
Leukaemias	9248.4	1.1	0.5	18	7.4	9
Liver cancer	5450.3	0.7	0.3	19	5.8	12

## Discussion

In this paper years of life lost were counted and analyzed by the method described by Christopher Murray and Alan Lopez in GBD 1990. It enabled us to compare the situation in Poland with other countries applying this methodology. It needs to be observed, however, that the 2010 Global Burden of Diseases, Injuries, and Risk Factors Study (GBD 2010) took into account certain epidemiological changes that occurred during the previous two decades and proposed certain modification in the methodology, which should be integrated in future research. Given the progress in extending life expectancy in the last 20 years, for the GBD 2010 study, it was decided to use the same reference standard for males and females and to use a life table based on the lowest observed death rate for each age group in countries of more than 5 million in population. The new GBD 2010 reference life table has a life expectancy at birth of 86.0 years for males and females. Taking into consideration many arguments for and against discounting future health and age-weighting in burden of disease measurement, it was decided that YLLs are computed with no discounting of future health and no age-weights [13].

The life lost years coefficients for the inhabitants of Poland decline systematically. In 1999, which is often selected as the point of departure for epidemiological analyses in Poland because of a major administrative reform of the country, the SEYLL_p_ measure amounted to 73.9 per 1,000 inhabitants (97.3 per 1,000 males and 51.8 per 1000 females), which means they were higher than in 2011 by approximately 25%.

According to research conducted by Marshall, if years of life lost per death is calculated to be about 9–10 years, it is not out of the ordinary and means that the age at death is congruent to the model life tables for Western developed nations (MLTW) age structure [11,12]. The number of years of life lost amounted to 6.0 per single death in Poland in 2011, which is lower than norms. It is worth noting that while in Marshall’s studies there are only slight differences between men and women, this differential in Poland is quite substantial (7.1 per 1 dead man and 4.7 per one dead woman).

The structure of the three broad cause groups of the SEYLL measure within Poland resembles that seen in other European countries [14-17]. Diseases from Group II, i.e. chronic non-communicable diseases, undoubtedly contribute to the greatest number of lost years of life. Diseases from Group I, i.e. communicable diseases and maternal, perinatal and nutritional disorders, cause fewer lost years of life. The most visible differences can be observed in Group III, i.e. injuries. Of European countries, Poland and other Eastern and Central European countries,together with Finland, Portugal and France, experience the greatest number of years of lost life due to injuries [18]. Injuries caused 10.1%of total lost yearsof life in Spain and 5.3% in Germany,but as much as 16.0%in Poland. The difference which puts Poland in such a negative position is the high number of lost years of life experienced by males. The SEYLL_p_ measure was 16.1 per 1,000 malesfor Poland compared with 7.3 per 1,000 malesfor Spain. Regarding women, the difference was much smaller: 2.9 per 1,000 females in Poland and 2.1 per 1,000 females in Spain.

A detailed analysis for the Lodz province, one of 16 provinces in Poland, confirmed that external causes of death, suicide in particular, represent a serious epidemiological problem, particularly for males. In 1999–2010, the number of years of life lost by males due to suicide systematically increased by 1.7% a year [19]. Although a decreasing tendency was observed in the death rate associated with the second most common factor, i.e. injuries, or traffic accidents, the rate still remains one of the highest in Europe. In 2011, higher SDR values were observed only in Romania, Greece and Latvia [8]. Traffic accidents contribute to the greatest number of deaths in people below the age of 25, which results in a great number of years of lost life. This loss of years mainly affects males, as 75% of people involved in traffic accidents are men. The widespread use of motor vehicles and motorbikescontributes to these statistics, especially those vehicles whose drivers often get involved in accidents, engage in drink-driving and exceed speed limits [19].

Of the Group II causes, non-communicable diseases, cardiovascular diseases and malignant neoplasms contribute to the greatest number of years of life lost, representing 42% and 37% of total years respectively. Since 1991, the position of cardiovascular diseases as the main cause of death in Poland has been systematically eroded [20,21]. Ischemic heart disease was found to have the greatest individual decrease as a cause of lost years in the Lodz Province [22]. However, it should be pointed out that the SEYLL_p_ measure due to this cause is still the highest of all single disease entities in males and the second highest in females.

However, heart failure is characterized by a reverse trend. The number of years of life lost due to this cause is growing and in 2011, it was in 6^th^ position for males and 3^rd^ position for females in Lodz [22]. This implies a relationship between mortality due to ischemic heart disease and heart failure, with the latter being a final stage of cardiac damage, which itself is a consequence of various diseases. Progress in the treatment of acute coronary syndrome has improved prognosis in acute myocardial infarction, and significantly reduced mortality. However, although many people survive infarction, extensive cardiac damage gradually occurs which leads to heart failure. Paradoxically, improvements in diagnostics and treatment of cardiovascular diseases, particularly ischemic heart disease and arterial hypertension, lead to an increase in morbidity of cardiac failure.

In the group of malignant neoplasms, lung cancer contributes to a great number of years of life lost. Although in Poland, as can be seen in Western Europe, the incidence of lung cancer in men has been decreasing, a reverse trend can be observed for women [23-27]. Despite its diminishing tendency, the number years of life lost due to this cause is still very high in males, occupying 2^nd^ position for single disease entities. For women, the trend has been systematically growing for some years, with the number of years of life lost in Poland in 2011 due to lung cancer (2.5 years per 1,000 females) being higher than the number of years of life lost due to breast cancer. Although nipple malignancies no longer occupy the first position, they nevertheless represent a serious life-threatening factor for females. Mortality due to nipple cancer is significantly more negative for younger women living in Poland than those living in other European countries [28], and forecasts indicate that it will increase over the forthcoming decades [29].

Regarding the remaining diseases in group II, liver cirrhosis is the third death cause leading to the highest number of life years lost per 1 person deceased due to a given cause. Mortality due liver cirrhosis is undoubtedly related to alcohol consumption. A Central Statistical Office study in 2009 showed that the average alcohol consumption calculated in pure alcohol amounted in Poland to 10.1 liters per person being 15 years old and more, which was slightly below the European average of 10.7 liters. However, the structure of alcohol consumption in Poland is unfavourable with above average consumption of strong alcohols and beer (respectively 3.76 l and 5.36 l) compared to the European Union (2.37 and 4.23 liters), while the consumption of wine in Poland (0.99 l) is one of the lowest all over Europe (where the average is 3.89 liters per person aged 15 and more [30]. In Spain, where the annual consumption of alcohol is higher than in Poland (11.4 liters), but win is much more important in the structure of alcohol consumption, SEYLL_p_ coefficients due to liver cirrhosis amount to 1.6 per 1,000 males and 0.5 per 1,000 females, considerably less than in Poland [15].

Communicable diseases, as well as maternal, perinatal and nutritional disorders, contribute a relatively small number of years of life lost, both in Poland and in other developed European countries (5.3% of the total value of the SEYLL measure, with the SEYLL_p_ equal to 3.1 per 1,000 inhabitants); in comparison, diseases from Group III contributed to 12.7% of lost years of life in Hong Kong, and the SEYLL measure was 11.8 per 1,000 inhabitants [31].

### Limitations of the study

As the reliability of statistical analysis performed on the basis of deaths depends to the largest extent on the correct identification of the underlying cause of death, in particular among the elderly, certain changes were introduced in Poland in 2009. In order to standardize the recording of the cause of death, which are subject to further statistical analysis, it was determined that the doctor who states the death should be responsible for completing the death card with the underlying, secondary and direct causes of death, whereas qualified teams of doctors are responsible for coding these causes of death according to the ICD-10 classification. In addition, the duties of a dozen regional statistical offices were taken over by the Central Statistical Office of Poland. Unfortunately, the relatively short time that the new system of processing data on deaths has been operating prevents its evaluation. In future, it would be useful to compare the registered causes of death in the Central Statistical Office with actual medical documentation concerning the history of the disease in a randomly selected sample.

## Conclusions

The analysis of standard expected years of life lost is aimed at emphasizing not only the social but also the economic aspect of the loss resulting from premature mortality. A further decrease in mortality due to cardiovascular diseases, whose incidence is extremely high, may prove beneficial as it would most effectively reduce the number of premature deaths. Moreover, from the economic point of view, the most effective preventative activities are those which aim at reducing the greatest number of years of life lost at a productive age per one death due to a particular reason, i.e. road traffic accidents, suicides and liver cirrhosis.
